# Suicide among patients with cancer: a call to action for researchers and clinical caregivers

**DOI:** 10.1002/ctm2.946

**Published:** 2022-06-29

**Authors:** Hansjörg Baurecht, Michael Heinrich, Peter M. Kreuzer, Berthold Langguth, Luisa Hofmann, Michael F. Leitzmann, Corinna Seliger

**Affiliations:** ^1^ Department of Epidemiology and Preventive Medicine University of Regensburg Regensburg Germany; ^2^ Department of Psychiatry and Psychotherapy University of Regensburg Regensburg Germany; ^3^ Department of Neurology University Hospital Heidelberg Heidelberg Germany

1

For a patient, the diagnosis of cancer represents a life‐changing event and inherently causes severe psychosocial distress, which under certain unfavourable circumstances may result in suicidal ideations and suicidal action. In addition to the primary diagnosis, a number of related factors such as anxiety, pain, fatigue, loss of perspectives, treatment‐related adverse events and lack of coping strategies may induce suicidal thoughts and behaviors.^1^ Suicides are a huge burden for family members, friends, as well as caregivers and society as a whole to cope with, as the loss of a beloved person goes far beyond the sole clinical case of a cancer patient.

A recent comprehensive systematic review and meta‐analysis quantified the increased suicide mortality among cancer patients compared to the general population and identified a series of relevant risk factors.^2^ Summarizing 62 studies including 46 952 813 patients revealed an almost two‐fold elevated suicide mortality rate among cancer patients and identified poor prognosis, late cancer stage, the first year after diagnosis and living in the US as high risk factors, while intermediate prognosis, early cancer stage and non‐sex‐specific cancers were reported as intermediate‐level risk factors.^2^

**FIGURE 1:**
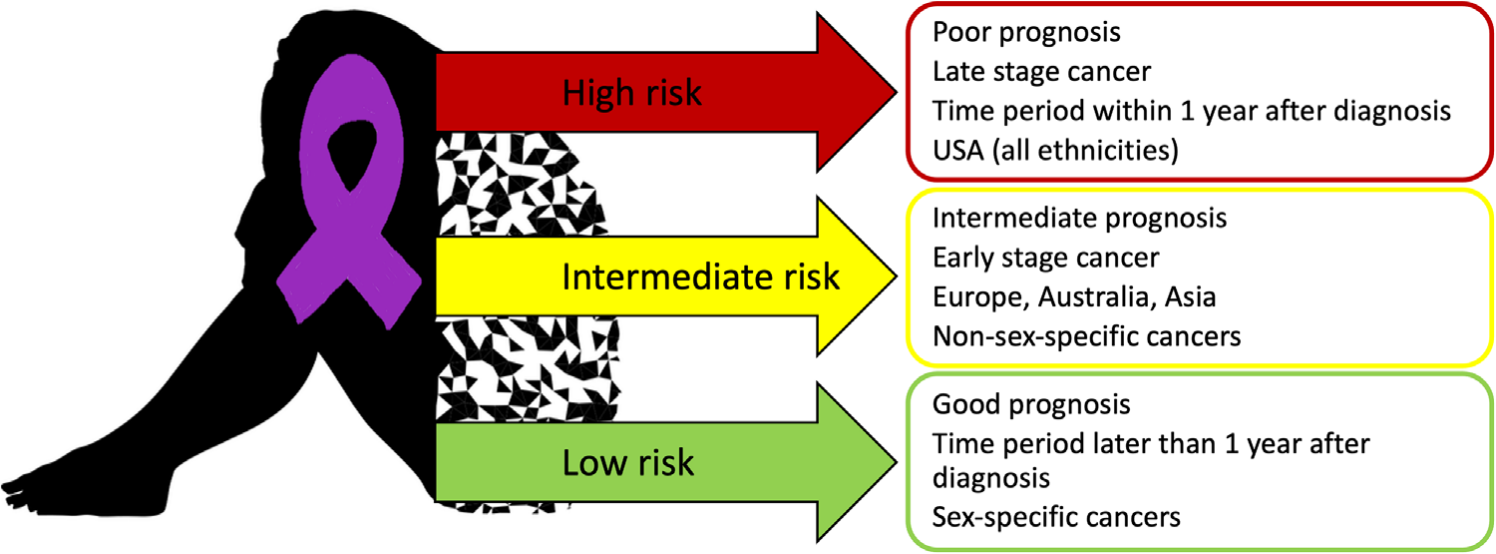
Suicide risk factors by grade of standard mortality ratio (SMR) among patients with cancer. Low risk was defined as SMR=1.50; intermediate risk as SMR 1.51–1.99; high risk SMR=2.00

The highest suicide mortality risk was identified for patients with cancer suffering from a poor prognosis, with a more than 3.5‐fold increased suicide risk compared to the general population.^2^ Correspondingly, advanced tumour stages were associated with a more than 3‐fold increased suicide risk (Figure 1).^2^ However, apart from these cancer‐related factors, a number of influencing factors unrelated to the characteristics of cancer were discussed: hopelessness, the expectation of aggressive therapies or severe cancer‐related symptoms may drive suicidality among patients with aggressive and advanced cancers. It is still unclear whether a patient's will for self‐autonomy or severe depressive symptoms cause suicidality among those patients.^3^ These accompanying factors are usually addressed by psycho‐oncology, which typically has a clear focus on psychotherapy rather than psychopharmacology. This may be based on the assumption that psychological reactions to a life‐changing event like cancer diagnosis require “psychological” treatment.

An important risk factor related to cancer diagnosis and prognosis is the time since diagnosis. Patients who survive the first year after diagnosis have a significantly lower suicide risk, although risk is still elevated compared to the general population.^2^ A cancer diagnosis may be perceived as trauma, which, in turn, may result in typical acute and long‐term consequences.^4^ Many patients experience peritraumatic distress, derealisation or dissociation during the medical consultation in which the cancer diagnosis is communicated.^5^ Acute traumatic reactions such as anger, shame, guilt, horror, heightened physiological arousal (racing heartbeat, sweating, shaking, etc.) and altered cognitions such as recurring fear attacks, racing thoughts, inability to focus or concentrate, avoidance, etc., have negative effects on the quality of life.^4^ These reactions may also directly or indirectly influence treatment success, for example, by reduced therapy compliance. The high suicide rate within the first year after diagnosis can probably be explained by an interplay of (objective or perceived) prognostic perspectives, the tumour stage as well as social and personal consequences of a life‐changing event. Life‐changing events were reported in 80% of recent suicides.^6^ It may be assumed that the reported elevated risk for committing suicide in cancer patients represents the “tip of the iceberg”, probably reflecting substantially increased psychological strain among these patients as a whole.

This systematic review also reports that the highest suicide rates worldwide among cancer patients are found in the USA, with rates 1.5 times higher than in European countries.^2^ This finding was opposed to similar suicide rates observed in the general populations. In contrast to many European countries, the USA lacks a universal health care system with easy access for a broad majority of the population.^7^ Therefore, cancer treatment may represent an enormous financial burden and additional psycho‐oncological care may not be affordable. Furthermore, access to firearms or cultural factors, such as a strong belief in autonomy may be other possible reasons.^2^ These psychosocial aspects with altered suicide risk depending on geographic location and potential fear of posing an economic burden for affected families definitely raise ethical questions especially (but not exclusively) with respect to assisted suicide.

Despite substantial advances in cancer treatment in the past years, improved cancer survival rates, and a clear focus on quality of life as a relevant outcome in cancer research, it seems astonishing that suicide among cancer patients is a widely neglected problem. The data of the review clearly show that suicide among cancer patients represents a highly relevant issue, but this situation seems to be insufficiently addressed both in research and clinical care thus far. Moreover, there might be an attitude among both health professionals and the general populations to consider suicidal ruminations in a cancer patient as a comprehensible or perhaps even unavoidable consequence of a severe disease with a poor prognosis. Such a mindset might result in therapeutic nihilism, which is definitely inappropriate.

The authors hope to stimulate further research about related symptoms and disorders (e.g., depression, anxiety, post‐traumatic stress disorder), relevant psychosocial factors, and the development of specific therapeutic interventions.

Physicians and other caregivers should be aware of those risks to integrate specialized psycho‐oncologists and psychiatrists more prominently and as early as possible in a holistic therapeutic approach not only to prevent suicides, but also to improve the quality of life of affected patients.
